# Editorial

**Published:** 1855-04

**Authors:** 


### Editorial.

### CONTINUOUS GUM WORK.

Dr. B. A. Satterthwaite, of Lima, Ohio, has recently sent us a fine specimen of what might really be called continuous gum. The entire palital arch, is covered by continuous gum. Throwing aside as Dr. Loomis recommends metallic plates of every kind The teeth, however, which Dr. Satterthwaite uses in his work is not carved in the material itself; but like continuous gum-work, they are single teeth, incorporated in the body before baking, and appear to be without back straps or any thing of the kind, indeed there is nothing to fasten back straps to. D. S. says, he is satisfied he can make such work to fit the mouth, perhaps equal to gold plate. Still as with Dr. Loomis's plan, we cannot think they will prove sufficiently strong; if so, we see no reason why Dr, Allen's method should not be abundantly so, for we have the additional strength of the metallic plate, also an outer and inner rim of the same. Dr. S., we would further remark, is still urging on his experiments in the manufacture of teeth, and from a set of gum teeth which he recently sent us, we infer that his ultimate design, is to carry off a big premium before long; these teeth bear the marks of great strength, naturalness of shape, and transparency of body, his gum is also finely colored. We know not to what extent he is manufacturing, yet we feel assured he is laboring to excel.

We are asked by two or three of our subscribers, do you still use Dr. Allen's continuous gum work? We answer, we do. There are many cases where we think it fulfills a better purpose than any thing else. There are also many cases where we prefer gold work and single teeth. We believe the latter will bear more hard falls and usage than the former. Yet when the other is put up as we now do it, rimmed inside and outside, and the body well fused, and let cool in the furnace, it is sufficiently strong for most of cases when proper care is taken, and it is certainly when neatly executed, beautiful, sweet and cleanly. Yet when imperfect it may become as offensive as any other. Time is testing its utility; and experience and American ingenuity, we hope, will make it more perfect, and before long we hope the court will decide on right and priority of discovery.

### OHIO COLLEGE OF DENTAL SURGERY.

The commencement exercises of this Institution, took place on 23d Feb. last. It was one of the most interesting occasions that we have ever had in connection with the school.

The degrees were conferred by Rev. Dr. Aydelotte—valedictory to the class by ourself, and reply by H. Munro, D.D.S. one of the graduates. These are published by the special request of the class.

The following gentlemen composed the graduating class :

J. DOUTHETT, Xenia, Ohio
H. MUNRO, Lebanon, Ky.
E. J. JONES, Dayton, Ohio.
R. C. REID, Xenia, Ohio.
W. A. CORNELIUS, Covington, Ky.

Owing to Prof. T. Wood's connection with the Medical College of Ohio, as Professor of Anatomy, he felt under the necessity of resigning his chair in the school. And the Association of the Ohio Dental College recommended that a chair of Chemistry and Metallurgy be created. Also that the chair of Practical Dentistry, &c., be changed to that of Institutes of Dental Science. Dr. C. B. Chapman, was recommended to the Board of Trustees, for the Chair of Anatomy and Physiology, Dr. George Watt, for that of Chemistry and Metallurgy, and ourself for that of Institutes of Dental Science.

The Trustees of the Institution, held a meeting this day, and approved and confirmed these changes, so that we are again organized, and ready for the duties assigned us. The Faculty now stand as follows:

*Institutes of Dental Science ;* JAMES TAYLOR, M.D. D.D.S.
*Anatomy and Physiology ;* C. B. CHAPMAN, M. D.
*Pathology and Therapeutics ;* T. B. SMITH, M. D.
*Operative Dentistry ;* J. TAFT, D.D.S.
*Mechanical Dentistry ;* H. R. SMITH, M.D., D.D.S.
*Chemistry and Metallurgy ;* GEORGE WATT, M.D. D.D.S.

Drs. Chapman and Watt, are both tried lecturers, and are both eminently fitted for the Chairs assigned them. It is with regret that we part with Dr Wood, who has so ably filled the Chair of Anatomy in our school, and hope his success will be commensurate with his talents, where his labors are now required.

---

TO THE DENTAL PROFESSION.—We, the undersigned Dentists of Philadelphia, take pleasure in recommending to the notice of the members of the Dental Profession an Improved method of MOUNTING TEETH on Atmospheric Plates, either gold or platina, invented by Dr. J. K. Rickey. We believe that the Dentist, by using this method, can make a perfect atmospheric plate, without the great difficulty they have heretofore labored under, namely, the warping or spring of plates, which this entirely overcomes.

A. R. JOHNSON,

S. DILLINGHAM,

HENRY AVERY,

WM. R. WHITE,

WM. J. A. BIRKEY,

ELY PARRY,

CHARLES A. DU BOUCHET,

JAS. M. HARRIS,

T. W. WALKER,

CHAS. C. PARRY,

J. W. VANOSTEN,

C. L. MUNUS,

T. RUSHING DAVIS,

G. E. MURRAY,

JOS. E. MILHENNEY & SON,

T. L. BUCKINGHAM,

C. S. HOOKER,

CALEB I. DIXON,

DAVID ROBERTS,

J. BIRKEY,

PHILADELPHIA, January 27th, 1855.

The above card we publish by request of Dr. J. K. Rickey, that the Profession may know the estimate which many of the Profession in Philadelphia, place on Dr. R.'s method of mounting teeth on atmospheric plates. We have tried the plan in several instances, with good results. The idea is to avoid the springing of a plate which has once been well adapted to the mouth. To accomplish this, the teeth are soldered to a narrow plate only a little wider than the base of the teeth, and this is riveted to the main plate. The latter, therefore, need not, and should not be annealed after swedging up to fit the mouth, It is thus left stiffer, and a thinner plate answers the purpose. The riveting of the two together adds strength and stiffness to the operation. If the main plate is rimmed, a solution of gutta percha in chloroform is used around the base of the teeth, &c. to make all air tight and sweet.

Dr. R. uses either gold or platina, sometimes platina for the narrow plate and gold for the main plate, when this is done, before the teeth is soldered on a layer of gold foil, may be united to it by fusing the gold.

Dr. Rickey remarks in a note to us, that platina plate No. 31, Stubs gague, when used for both plates, is sufficiently stiff and strong when riveted together, the readers of the Register know that Dr. Rickey has obtained a patent on his improvement. Some of the Profession we are aware deny the priority of Dr. R.'s improvement. Many years since we saw teeth riveted to the plate by Dr. Rostaing of this city ; but this was done to avoid cracking of the teeth and the trouble of soldering. He was a perfect adept at such shifts to avoid the use of the blow pipe, and yet had more kinds of blow pipes than any man we ever saw.

There are a great many things which appear to require the shield of a patent to bring them into use ; as soon as it thus known, hundreds begin to divulge the long concealed secret. So that while we regard the principle which seeks the benefit of the patent law as unprofessional, we regard the other, that of concealment worse. There are three sets of persons who make up our Profession; one who know and keep every thing; another who think the patent office the best *standard* work in the Profession, and the third, those who regard our periodicals as the very channels through which to disseminate useful knowledge.

The improvement which Dr. Rickey claims and has patented, is we know entirely practical, and possesses much merit. Dr. R. is located at Keokuk, Iowa.

---

### JONES, WHITE AND M'CURDY'S TEETH.

It will be seen by reference to the proceedings of the Miss. Val. Ass., that these gentlemen's teeth received especial commendation. Their recent manufucture deserve some special notice, owing to some improvement made, not that we suppose their teeth could be much improved, as it regards strength and life-like appearance ; but there are such a variety of shapes and patterns needed in practice, that every addition in this respect, we regard as an improvement. It is with pleasure we see these gentlemen striving to meet every want of the Profession, even in this respect. It shows a determination not to be excelled or driven from the market, by other rival establishments who are also making every effort for a share of patronage. The term "Eye Bicuspids," which our friend Dr. J. M. Brown gives to their new style of superior bicuspids, almost describes one of their improvements which we regard as very useful. It is simply these teeth made with a short inner cusp. We hope they will give us more of these of a smaller class of teeth. They are much prized and much needed.

The upper incisors and cuspids, with a shoulder on their lingual face, are also at times, admirably suited to meet exigencies in practice. They do away with the necessity of making gold standards for the under teeth to strike upon, when partial sets are wanted, and the molars are gone these are wanted.

Their curved gum teeth are also an improvement, when the alveolar arch is prominent. These things with their decided improvement in gum, constitute much for commendation.

### DENTAL ADVERTISER.

This circular periodical to the Profession, by Dr. J. M. Brown of this city, is again before us. It shows up so well the labors of this gentleman to meet the wants of the Profession in the West and South, that we feel there is but little need on our part to attempt to post up the Profession in this department. We feel that if the Dr. could attend to our operations as well as the "furnishing," we might give the whole into his hands.

We believe that his great pride is to keep the best "furnishing establishment" in the United States ; and from the extent and increase of his business, we judge that he fulfills about what he purposes. Besides the best of instruments from the eastern manufacturers, he keeps Mr. Sherwood with quite a number of hands continually employed to fill orders for this gentleman's superior forceps, pluggers, &c. &c. We notice in his establishment a new *saliva pump*. This is a neat and convenient article for getting rid of the saliva during the plugging of the under teeth. Among other articles to be had at his establishment, can be had Jones, White & McCurdy's teeth ; Oram and Armstrong's teeth ; Alcock's teeth ; material for continuous gum work ; gold ; platinum and silver plate ; solder, &c. Auroplastic base, for artificial teeth ; gold foil of different manufactures ; tin foil of different manufactures ; rolling mills ; operating chairs ; all kinds of dental instruments ; all the different preparations of crystal gold, sponge, and prepared gold for filling teeth, &c.

### DENTAL MONITOR.

This is the title of a new Quarterly, devoted to the special wants of the public who need the services of the Dentist. Its style and typographical appearance is attractive, and being designed for the general reader, its pages are filled with that which would more likely please the patient waiting to be operated upon than the operator.

To what extent such publications will command the attention and patronage of the public is yet to be tested. Such a work might be made to embody a great deal of useful matter, which would exert a very beneficial effect on the community, dissipating many false notions as it regards dental operations, diseases of the teeth, &c.

We presume, however, that for a while at least, its circulation will of necessity be principally among the members of the Profession, who wish to keep such a work on their table for the use of their patients. In the present number, we confess we like the dental department better than the miscellaneous or juvenile, and especially, the comico-poetic,

These things, however, may do to please the juveniles. The work is a twenty-four page octavo, published quarterly, at the low price of 50 cents per annum; and is edited and published by J. G. Ambler, M. D., D.D.S. New York.

### THE FORCEP.

This is the title of a new quarterly Journal, devoted to the advancement of Dental Science, published by the New York Teeth Manufacturing Company. It is filled with that which relates directly to the Profession, original and selected articles.

The main object appears to be a medium of advertising for the company's teeth, &c. The Profession may, however, be equally benefitted.

### DENTAL OBTURATOR.

This *closing* up of our new publications is to be issued 1st of May, and is designed to be strictly Professional and meet the wants of our Southern brethren, as a medium for the interchange of thought and concentration of effort for the advancement of Dental Science. From the talent and enterprize which will be brought to bear upon it, we have no doubt it will be a valuable acquisition to our periodical literature. It will be edited by Dr. J. S. Clark, of New Orleans, a gentleman well known in the Profession as an able writer and skilful operator. We bespeak for it an extensive circulation.

### MISSISSIPPI VALLEY ASSOCIATION OF DENTAL SURGEONS.

This society held its annual meeting on the 23d, 24th and 25th of February last, and the sessions were of much interest. The action of the society in relation to prize essays, and extracting instruments, will be seen by reference to the proceedings, which we have published in the present number.

The prize of $100 for the best essay, was awarded to Dr. George Watt, of Xenia, Ohio; and the premium of $50, to Messrs. Sherwood and Brown, for the best extracting instruments presented.

### OSSEOS UNION OF TEETH.

We are in receipt of a beautiful specimen of osseos union of two teeth, the lateral incisor and cuspid of the right side inferior jaw, of the deciduous set. The union is perfect throughout the entire length of the roots, and about one third of the enamel on their palital face. The enamel also at their necks on their labial face is united. They were removed from the mouth of a girl four years of age, by Dr. N. L. Slayton, of Madison, Ind., who will please accept our thanks for them. We shall put them in the College Museum.

### GOLD FOIL.

We call the attention of the Profession to the card of Mr. James Leslie, manufacturer of Dentists Gold Foil. Mr. Leslie has for a long time enjoyed an enviable reputation for the manufacture of gold foil in this city. He is now devoting special attention to this branch of his business. We feel assured that the quality of his foil will give satisfaction to the Profession, and having somewhat reduced its price, he solicits a share of patronage. Orders accompanied with cash will be promptly attended to.

### SUBSCRIBERS TO THE REGISTER.

We have taken special pains to revise our list of subscribers, and would be glad if any have not received all the numbers, to have them inform us of the fact, and we will cheerfully supply the loss. We are satisfied that in many cases, even when mailed they may never reach. In a few instances, subscribers have changed their location and not informed us of the fact. This gives us trouble, and we would be glad if such would always let us know.

We have pretty generally sent bills to those indebted, and now return our acknowledgements for the promptness with which many have responded. There are still, however, many to be heard from, and we hope they will not forget their bills—our *Printer* never forgets his.

We have been almost as much gratified at the kind feeling expressed in many letters, as with the remittance itself. We had intended to have given a few extracts from some of these letters, to contrast the sentiment received from two or three, who, because they had not *ordered* the Register, (*yet had been receiving it*), flared up considerably, showing more gas than Professional sense. We may refer to these some other time. In two or three instances mistakes have occurred in accounts. This has more frequently occurred, however, from the fact that most pay at the end of the year and not in advance, as they supposed.

### ENLARGEMENT OE THE REGISTER.

We have been requested by the Mississippi Association, to enlarge the Register. Our readers will perceive by comparing the first and second volumes with the present, that it is now nearly double its former size, and yet the price is the same. We hope to be able to enlarge the Register with the beginning of next volume, to 112 pages, and shall then issue it at $3 per year. This appears to be the wish of many of the Profession. And unless the plan is generally dissented from, between this and October next, we shall carry it into effect.

### AMERICAN SOCIETY OF DENTAL SURGEONS.

It will be seen by the following note, from Dr. E. Townsend, President of the American Society, to Dr. A. S. Talbert, President of the Miss. Val. Ass. that we shall have the pleasure of greeting the members of the A. S. on the 8th of May next, at 10 A. M. We hope the Profession generally, will visit our city at that time, and participate in the erjoyment which such meetings afford.

It will be seen also, that the Miss. Ass. hold a special meeting the day before. We presume the prize essay will then be finally acted on, and ordered for publication.

PHILADELPHIA, March 2d. 1855.

DR. TALBERT,—DEAR SIR :—Your favor of the 26th has just come to hand. In reply, The American Society of Dental Surgeons will convene on the second Tuesday, 8th of May, at 10 A. M. in the Ohio Dental College, which has been kindly offered for the purpose.

I may say in conclusion, we shall be very happy to welcome the members of Miss. Val. Ass. of Dental Surgeons, as many as may honor us with their company.

Very respectfully, yours,

E. TOWNSEND.

There will be a special meeting of the Mississippi Valley Association of Dental Surgery, held in the lecture room of the Ohio College of Dental Surgeons, on Monday the seventh day of May next at 4 o'clock, P. M. A full attendance is desired.

A. S. TALBERT,

Pres. Miss. Val. Ass. D. S.

LEXINGTON, March 5th, 1855.

OBITUARY.

We are pained to announce the recent decease of Dr. W. R. Scott, of Raleigh N. C. Dr. Scott has for years enjoyed an enviable reputation as an excellent operator and accomplished gentleman. We have also to notice the decease of Dr. M. K. Bridges, of Brooklyn, N. Y. Dr. Bridges was one of our oldest operators and always sustained a fine character as a dentist and gentleman. We feel the loss of such men in the profession.

ERRATA.

The following errors occur in Dr. C. T. Cushman's article, entitled, "Practica Thoughts on Tooth Drawing," published in the last number of the Register.

p. 101, Text of Desirabode, read practised, for "practical."
p. 101, Next line, read extraction for "evtraction."
p. 102, 10th line, read unavoidably, for "invariably."
p. 104, 3d line, read tooth, for "teeth."
p. 105, 9th line from bottom, read Samson, for "Sampson."
p. 107, 3d line, substitute comma for period, after character.
p. 107, 13th line, insert its, after with.

